# *trans*-Dichloridobis{[4-(dimethyl­amino)­phen­yl]diphenyl­phosphane}palladium(II)

**DOI:** 10.1107/S1600536810042595

**Published:** 2010-10-23

**Authors:** Alfred Muller, Reinout Meijboom

**Affiliations:** a Research Centre for Synthesis and Catalysis, Department of Chemistry, University of Johannesburg, PO Box 524 Auckland Park, Johannesburg, 2006, South Africa

## Abstract

The title compound, *trans*-[PdCl_2_{PPh_2_(4-Me_2_NC_6_H_4_)}_2_], crystallizes with the Pd atom on a center of symmetry, resulting in a distorted *trans*-PdCl_2_P_2_ square-planar geometry. The Pd—P and Pd—Cl bond lengths are 2.3550 (7) and 2.2906 (7) Å, respectively. Some weak inter­actions are observed between the aromatic rings of adjacent mol­ecules, with an inter­planar distance between two π-stacked rings of 3.505 (3) Å. Intra- and intermolecular C—H⋯Cl hydrogen bonds also occur.

## Related literature

For a review on related compounds, see: Spessard & Miessler (1996 ▶). For related compounds, see: Burrow *et al.* (1994 ▶); DiMeglio *et al.* (1990 ▶); Edwards *et al.* (1998 ▶); Ferguson *et al.* (1982 ▶); Grushin *et al.* (1994 ▶); Kitano *et al.* (1983 ▶); Leznoff *et al.* (1999 ▶); Meij *et al.* (2003 ▶); Meijboom *et al.* (2006*a* ▶,*b* ▶); Meijboom & Omondi (2010 ▶). For the synthesis of the starting materials, see: Drew & Doyle (1990 ▶).
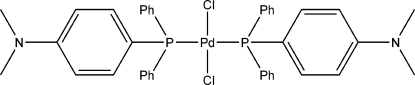


## Experimental

### 

#### Crystal data

[PdCl_2_(C_20_H_20_NP)_2_]
*M* *_r_* = 787.98
Triclinic, 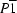

*a* = 9.9006 (16) Å
*b* = 9.9815 (15) Å
*c* = 10.4021 (14) Å
α = 86.291 (4)°
β = 69.135 (4)°
γ = 65.383 (4)°
*V* = 869.0 (2) Å^3^
*Z* = 1
Mo *K*α radiation
μ = 0.81 mm^−1^
*T* = 100 K
0.18 × 0.10 × 0.04 mm

#### Data collection

Bruker X8 APEXII 4K Kappa CCD diffractometer
Absorption correction: multi-scan (*SADABS*; Bruker; 2004 ▶) *T*_min_ = 0.870, *T*_max_ = 0.966
10917 measured reflections
4268 independent reflections
3545 reflections with *I* > 2σ(*I*)
*R*_int_ = 0.035

#### Refinement

*R*[*F* ^2^ > 2σ(*F* ^2^)] = 0.038
*wR*(*F* ^2^) = 0.096
*S* = 1.05
4268 reflections
216 parameters
H-atom parameters constrained
Δρ_max_ = 2.04 e Å^−3^
Δρ_min_ = −0.72 e Å^−3^

### 

Data collection: *APEX2* (Bruker, 2005 ▶); cell refinement: *SAINT-Plus* (Bruker, 2004 ▶); data reduction: *SAINT-Plus* and *XPREP* (Bruker, 2004 ▶); program(s) used to solve structure: *SIR97* (Altomare *et al.*, 1999 ▶); program(s) used to refine structure: *SHELXL97* (Sheldrick, 2008 ▶); molecular graphics: *DIAMOND* (Brandenburg & Putz, 2005 ▶); software used to prepare material for publication: *WinGX* (Farrugia, 1999 ▶).

## Supplementary Material

Crystal structure: contains datablocks global, I. DOI: 10.1107/S1600536810042595/pk2275sup1.cif

Structure factors: contains datablocks I. DOI: 10.1107/S1600536810042595/pk2275Isup2.hkl

Additional supplementary materials:  crystallographic information; 3D view; checkCIF report

## Figures and Tables

**Table 1:** Hydrogen-bond geometry (Å, °)

*D*—H⋯*A*	*D*—H	H⋯*A*	*D*⋯*A*	*D*—H⋯*A*
C25—H25⋯Cl^i^	0.95	2.79	3.710 (3)	163
C26—H26⋯Cl	0.95	2.74	3.159 (3)	108
C36—H36⋯Cl^ii^	0.95	2.77	3.529 (3)	138

Symmetry codes: (i) 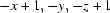; (ii) 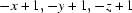.

**Fig. 1.**
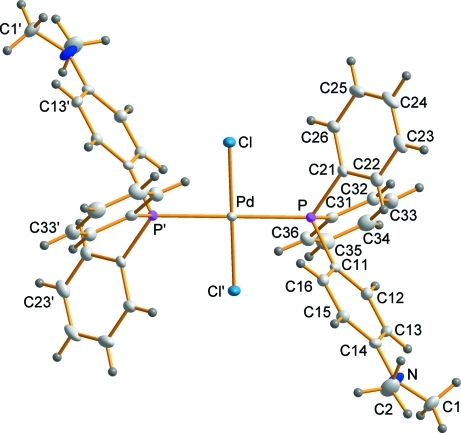
The structure (I), showing 50% probability displacement ellipsoids. For the C atoms, the first digit indicates ring number and the second digit indicates the position of the atom in the ring. Accented lettering indicate atoms generated by symmetry (1 - x,1 - y,1 - z).

## supplementary crystallographic information

### Comment

Transition metal complexes containing phosphine, arsine and stibine ligands are widely being investigated in various fields of organometallic chemistry (Spessard & Miessler, 1996). As part of a systematic investigation involving complexes with the general formula *trans*-[*MX*_2_(*L*)_2_] (*M* = Pt or Pd; *X* = halogen, Me, Ph; *L* = Group 15 donor ligand), crystals of the title compound were obtained.

[PdCl_2_(*L*)_2_] (*L* = tertiary phosphine, arsine or stibine) complexes can conveniently be prepared by the substitution of 1,5-cyclooctadiene (COD) from [PdCl_2_(COD)]. The title compound, *trans*-[PdCl_2_{PPh_2_(4-Me_2_NC_6_H_4_)}_2_], crystallizes in the triclinic spacegroup *P*1, with the Pd atom on a center of symmetry and each pair of equivalent ligands in a mutually *trans* orientation. The geometry is, therefore, slightly distorted square planar and the Pd atom is not elevated out of the coordinating atom plane. All angles in the coordination polyhedron are close to the ideal value of 90°, with P—Pd—Cl = 93.84 (2) and P—Pd—Cl^i^ = 86.16 (2)°. As required by the crystallographic symmetry, the P—Pd—P^i^ and Cl—Pd—Cl^i^ angles are 180°. Some weak intermolecular interactions were observed and are reported in Table 1.

The title compound compares well with other closely related Pd^II^ complexes from the literature containing two chloro and two tertiary phosphine ligands in a *trans* geometry. The title compound, having a Pd—Cl bond length of 2.2955 (13) Å and a Pd—P bond length of 2.3398 (12) Å, fits well into the typical range for complexes of this kind. It is notable that the title compound crystallized as an unsolvated complex, as these type of Pd^II^ complexes tend to crystallize as solvates (Meijboom & Omondi, 2010).

### Experimental

Dichloro(1,5-cyclooctadiene)palladium(II), [PdCl_2_(COD)], was prepared according to the literature procedure of Drew & Doyle (1990). A solution of diphenyl(4-dimethylaminophenyl)phosphine (0.2 mmol) in dichloromethane (2.0 cm^3^) was added to a solution of [PdCl_2_(COD)] (0.1 mmol) in dichloromethane (3.0 cm^3^). Slow evaporation of the solvent gave yellow crystals of the title compound.

### Refinement

The aromatic and methyl H atoms were placed in geometrically idealized positions (C—H = 0.95–0.98) and constrained to ride on their parent atoms with *U*_iso_(H) = 1.2*U*_eq_(C) for aromatic and *U*_iso_(H) = 1.5*U*_eq_(C) for methyl H atoms respectively. Methyl torsion angles were refined from electron density

### Crystal data

**Table:**

[PdCl_2_(C_20_H_20_NP)_2_]	*Z* = 1
*M**_r_* = 787.98	*F*(000) = 404
Triclinic, *P*1	*D*_x_ = 1.506 Mg m^−^^3^
Hall symbol: -P 1	Mo *K*α radiation, λ = 0.71073 Å
*a* = 9.9006 (16) Å	Cell parameters from 3426 reflections
*b* = 9.9815 (15) Å	θ = 2.3–28.1°
*c* = 10.4021 (14) Å	µ = 0.81 mm^−^^1^
α = 86.291 (4)°	*T* = 100 K
β = 69.135 (4)°	Plate, yellow
γ = 65.383 (4)°	0.18 × 0.1 × 0.04 mm
*V* = 869.0 (2) Å^3^	

### Data collection

**Table:**

Bruker X8 APEXII 4K Kappa CCD diffractometer	4268 independent reflections
Radiation source: fine-focus sealed tube	3545 reflections with *I* > 2σ(*I*)
graphite	*R*_int_ = 0.035
Detector resolution: 8.4 pixels mm^-1^	θ_max_ = 28.3°, θ_min_ = 2.3°
φ and ω scans	*h* = −13→12
Absorption correction: multi-scan (*SADABS*; Bruker; 2004)	*k* = −13→12
*T*_min_ = 0.870, *T*_max_ = 0.966	*l* = −13→13
10917 measured reflections	

### Refinement

**Table:**

Refinement on *F*^2^	0 restraints
Least-squares matrix: full	H-atom parameters constrained
*R*[*F*^2^ > 2σ(*F*^2^)] = 0.038	*w* = 1/[σ^2^(*F*_o_^2^) + (0.0458*P*)^2^ + 0.5716*P*] where *P* = (*F*_o_^2^ + 2*F*_c_^2^)/3
*wR*(*F*^2^) = 0.096	(Δ/σ)_max_ = 0.001
*S* = 1.05	Δρ_max_ = 2.04 e Å^−^^3^
4268 reflections	Δρ_min_ = −0.72 e Å^−^^3^
216 parameters	

### Special details

**Table:**

Experimental. The intensity data was collected on a Bruker X8 Apex II 4 K Kappa CCD diffractometer using an exposure time of 50 s/frame. A total of 604 frames were collected with a frame width of 0.5° covering up to θ = 28.28° with 99.0% completeness accomplished.
Geometry. All e.s.d.'s (except the e.s.d. in the dihedral angle between two l.s. planes) are estimated using the full covariance matrix. The cell e.s.d.'s are taken into account individually in the estimation of e.s.d.'s in distances, angles and torsion angles; correlations between e.s.d.'s in cell parameters are only used when they are defined by crystal symmetry. An approximate (isotropic) treatment of cell e.s.d.'s is used for estimating e.s.d.'s involving l.s. planes.

### Fractional atomic coordinates and isotropic or equivalent isotropic displacement parameters (Å^2^)

**Table:**

	*x*	*y*	*z*	*U*_iso_*/*U*_eq_	
Pd	0.5	0.5	0.5	0.01593 (10)	
Cl	0.62710 (9)	0.24815 (7)	0.44885 (7)	0.02265 (16)	
P	0.29234 (9)	0.49076 (7)	0.69623 (6)	0.01476 (15)	
C11	0.2478 (3)	0.6133 (3)	0.8410 (2)	0.0151 (5)	
C12	0.0928 (3)	0.7106 (3)	0.9190 (3)	0.0172 (5)	
H12	0.0075	0.7163	0.8936	0.021*	
C13	0.0600 (3)	0.7997 (3)	1.0331 (3)	0.0172 (5)	
H13	−0.047	0.8665	1.0836	0.021*	
C14	0.1827 (3)	0.7926 (3)	1.0749 (3)	0.0178 (6)	
C15	0.3404 (3)	0.6938 (3)	0.9942 (3)	0.0180 (6)	
H15	0.4266	0.6857	1.0195	0.022*	
C16	0.3700 (3)	0.6091 (3)	0.8791 (3)	0.0186 (6)	
H16	0.4771	0.5463	0.8245	0.022*	
N	0.1501 (3)	0.8796 (3)	1.1885 (3)	0.0266 (6)	
C1	−0.0146 (4)	0.9674 (3)	1.2752 (3)	0.0255 (6)	
H1A	−0.0691	1.0378	1.2204	0.038*	
H1B	−0.0181	1.0218	1.3523	0.038*	
H1C	−0.0679	0.902	1.3113	0.038*	
C2	0.2712 (4)	0.8525 (4)	1.2451 (3)	0.0322 (7)	
H2A	0.3102	0.75	1.2684	0.048*	
H2B	0.226	0.9202	1.3287	0.048*	
H2C	0.3597	0.8689	1.1768	0.048*	
C21	0.3219 (3)	0.3123 (3)	0.7667 (3)	0.0170 (5)	
C22	0.3088 (3)	0.2961 (3)	0.9042 (3)	0.0198 (6)	
H22	0.2843	0.3788	0.9627	0.024*	
C23	0.3316 (4)	0.1597 (3)	0.9563 (3)	0.0250 (6)	
H23	0.3219	0.1496	1.0502	0.03*	
C24	0.3682 (4)	0.0391 (3)	0.8717 (4)	0.0287 (7)	
H24	0.3867	−0.0547	0.9067	0.034*	
C25	0.3781 (4)	0.0548 (3)	0.7352 (3)	0.0264 (7)	
H25	0.4008	−0.0278	0.6776	0.032*	
C26	0.3549 (3)	0.1904 (3)	0.6835 (3)	0.0208 (6)	
H26	0.3616	0.2005	0.5902	0.025*	
C31	0.1021 (3)	0.5480 (3)	0.6741 (3)	0.0171 (5)	
C32	−0.0064 (4)	0.4905 (3)	0.7501 (3)	0.0206 (6)	
H32	0.0225	0.4163	0.8095	0.025*	
C33	−0.1543 (4)	0.5394 (3)	0.7404 (3)	0.0249 (6)	
H33	−0.2256	0.4979	0.792	0.03*	
C34	−0.1998 (4)	0.6491 (4)	0.6554 (3)	0.0275 (7)	
H34	−0.3023	0.6842	0.6495	0.033*	
C35	−0.0932 (4)	0.7064 (4)	0.5797 (3)	0.0290 (7)	
H35	−0.1235	0.7817	0.5217	0.035*	
C36	0.0567 (4)	0.6564 (3)	0.5868 (3)	0.0238 (6)	
H36	0.1289	0.6957	0.5323	0.029*	

### Atomic displacement parameters (Å^2^)

**Table:**

	*U*^11^	*U*^22^	*U*^33^	*U*^12^	*U*^13^	*U*^23^
Pd	0.02125 (18)	0.01026 (15)	0.01176 (14)	−0.00809 (12)	0.00111 (11)	−0.00095 (10)
Cl	0.0274 (4)	0.0115 (3)	0.0198 (3)	−0.0082 (3)	0.0023 (3)	−0.0020 (2)
P	0.0190 (4)	0.0113 (3)	0.0110 (3)	−0.0080 (3)	−0.0001 (3)	−0.0002 (2)
C11	0.0219 (15)	0.0096 (12)	0.0116 (11)	−0.0085 (11)	−0.0013 (10)	0.0007 (9)
C12	0.0181 (14)	0.0165 (13)	0.0155 (12)	−0.0089 (12)	−0.0025 (11)	0.0003 (10)
C13	0.0159 (14)	0.0130 (13)	0.0156 (12)	−0.0049 (11)	0.0011 (10)	−0.0024 (10)
C14	0.0227 (15)	0.0111 (13)	0.0165 (12)	−0.0079 (11)	−0.0025 (11)	−0.0005 (10)
C15	0.0171 (14)	0.0173 (14)	0.0186 (12)	−0.0079 (12)	−0.0041 (11)	−0.0005 (10)
C16	0.0186 (15)	0.0143 (13)	0.0177 (12)	−0.0071 (11)	−0.0003 (11)	0.0001 (10)
N	0.0215 (14)	0.0294 (14)	0.0234 (12)	−0.0073 (12)	−0.0032 (11)	−0.0139 (10)
C1	0.0247 (17)	0.0260 (16)	0.0175 (13)	−0.0061 (13)	−0.0026 (12)	−0.0067 (11)
C2	0.0291 (18)	0.039 (2)	0.0296 (16)	−0.0137 (16)	−0.0112 (14)	−0.0086 (14)
C21	0.0169 (14)	0.0135 (13)	0.0169 (12)	−0.0085 (11)	0.0001 (11)	0.0018 (10)
C22	0.0186 (15)	0.0200 (14)	0.0195 (13)	−0.0090 (12)	−0.0047 (11)	0.0037 (11)
C23	0.0216 (16)	0.0255 (16)	0.0295 (15)	−0.0125 (13)	−0.0096 (13)	0.0123 (13)
C24	0.0210 (16)	0.0168 (15)	0.0468 (19)	−0.0091 (13)	−0.0113 (14)	0.0144 (14)
C25	0.0216 (16)	0.0154 (14)	0.0377 (17)	−0.0096 (13)	−0.0032 (13)	−0.0012 (12)
C26	0.0206 (15)	0.0155 (14)	0.0225 (13)	−0.0090 (12)	−0.0016 (12)	0.0000 (11)
C31	0.0227 (15)	0.0137 (13)	0.0129 (11)	−0.0073 (11)	−0.0042 (11)	−0.0019 (10)
C32	0.0259 (16)	0.0189 (14)	0.0155 (12)	−0.0099 (13)	−0.0052 (11)	0.0024 (10)
C33	0.0282 (17)	0.0269 (16)	0.0216 (14)	−0.0152 (14)	−0.0069 (13)	0.0010 (12)
C34	0.0292 (18)	0.0280 (17)	0.0269 (15)	−0.0102 (14)	−0.0139 (13)	−0.0001 (13)
C35	0.0373 (19)	0.0247 (16)	0.0285 (15)	−0.0116 (15)	−0.0184 (15)	0.0074 (13)
C36	0.0331 (18)	0.0219 (15)	0.0183 (13)	−0.0150 (14)	−0.0077 (12)	0.0040 (11)

### Geometric parameters (Å, °)

**Table:**

Pd—Cl^i^	2.2906 (7)	C2—H2B	0.98
Pd—Cl	2.2906 (7)	C2—H2C	0.98
Pd—P^i^	2.3550 (7)	C21—C26	1.393 (4)
Pd—P	2.3550 (7)	C21—C22	1.395 (4)
P—C11	1.809 (3)	C22—C23	1.391 (4)
P—C31	1.823 (3)	C22—H22	0.95
P—C21	1.829 (3)	C23—C24	1.379 (5)
C11—C16	1.385 (4)	C23—H23	0.95
C11—C12	1.389 (4)	C24—C25	1.391 (5)
C12—C13	1.388 (4)	C24—H24	0.95
C12—H12	0.95	C25—C26	1.382 (4)
C13—C14	1.404 (4)	C25—H25	0.95
C13—H13	0.95	C26—H26	0.95
C14—N	1.372 (3)	C31—C36	1.398 (4)
C14—C15	1.416 (4)	C31—C32	1.400 (4)
C15—C16	1.382 (4)	C32—C33	1.377 (4)
C15—H15	0.95	C32—H32	0.95
C16—H16	0.95	C33—C34	1.390 (4)
N—C2	1.438 (4)	C33—H33	0.95
N—C1	1.451 (4)	C34—C35	1.384 (5)
C1—H1A	0.98	C34—H34	0.95
C1—H1B	0.98	C35—C36	1.383 (4)
C1—H1C	0.98	C35—H35	0.95
C2—H2A	0.98	C36—H36	0.95
			
Cl^i^—Pd—Cl	180.0000 (10)	H2A—C2—H2B	109.5
Cl^i^—Pd—P^i^	93.84 (2)	N—C2—H2C	109.5
Cl—Pd—P^i^	86.16 (2)	H2A—C2—H2C	109.5
Cl^i^—Pd—P	86.16 (2)	H2B—C2—H2C	109.5
Cl—Pd—P	93.84 (2)	C26—C21—C22	118.8 (2)
P^i^—Pd—P	180.0000 (10)	C26—C21—P	120.1 (2)
C11—P—C31	104.37 (12)	C22—C21—P	121.1 (2)
C11—P—C21	104.02 (12)	C23—C22—C21	120.4 (3)
C31—P—C21	102.69 (13)	C23—C22—H22	119.8
C11—P—Pd	111.57 (9)	C21—C22—H22	119.8
C31—P—Pd	114.91 (8)	C24—C23—C22	120.1 (3)
C21—P—Pd	117.81 (9)	C24—C23—H23	120
C16—C11—C12	118.0 (2)	C22—C23—H23	120
C16—C11—P	119.9 (2)	C23—C24—C25	120.0 (3)
C12—C11—P	122.1 (2)	C23—C24—H24	120
C13—C12—C11	121.4 (3)	C25—C24—H24	120
C13—C12—H12	119.3	C26—C25—C24	120.0 (3)
C11—C12—H12	119.3	C26—C25—H25	120
C12—C13—C14	120.9 (3)	C24—C25—H25	120
C12—C13—H13	119.6	C25—C26—C21	120.7 (3)
C14—C13—H13	119.6	C25—C26—H26	119.7
N—C14—C13	120.9 (3)	C21—C26—H26	119.7
N—C14—C15	121.8 (3)	C36—C31—C32	118.2 (3)
C13—C14—C15	117.3 (2)	C36—C31—P	121.0 (2)
C16—C15—C14	120.5 (3)	C32—C31—P	120.7 (2)
C16—C15—H15	119.7	C33—C32—C31	121.2 (3)
C14—C15—H15	119.7	C33—C32—H32	119.4
C15—C16—C11	121.8 (3)	C31—C32—H32	119.4
C15—C16—H16	119.1	C32—C33—C34	120.3 (3)
C11—C16—H16	119.1	C32—C33—H33	119.8
C14—N—C2	120.2 (3)	C34—C33—H33	119.8
C14—N—C1	119.3 (3)	C35—C34—C33	118.8 (3)
C2—N—C1	118.0 (2)	C35—C34—H34	120.6
N—C1—H1A	109.5	C33—C34—H34	120.6
N—C1—H1B	109.5	C36—C35—C34	121.4 (3)
H1A—C1—H1B	109.5	C36—C35—H35	119.3
N—C1—H1C	109.5	C34—C35—H35	119.3
H1A—C1—H1C	109.5	C35—C36—C31	120.0 (3)
H1B—C1—H1C	109.5	C35—C36—H36	120
N—C2—H2A	109.5	C31—C36—H36	120
N—C2—H2B	109.5		
			
Cl^i^—Pd—P—C11	−44.26 (10)	C31—P—C21—C26	69.8 (3)
Cl—Pd—P—C11	135.74 (10)	Pd—P—C21—C26	−57.5 (3)
Cl^i^—Pd—P—C31	74.28 (10)	C11—P—C21—C22	−0.3 (3)
Cl—Pd—P—C31	−105.72 (10)	C31—P—C21—C22	−108.9 (2)
Cl^i^—Pd—P—C21	−164.46 (11)	Pd—P—C21—C22	123.7 (2)
Cl—Pd—P—C21	15.54 (11)	C26—C21—C22—C23	1.2 (4)
C31—P—C11—C16	−174.3 (2)	P—C21—C22—C23	180.0 (2)
C21—P—C11—C16	78.4 (2)	C21—C22—C23—C24	0.4 (4)
Pd—P—C11—C16	−49.7 (2)	C22—C23—C24—C25	−1.8 (5)
C31—P—C11—C12	7.3 (2)	C23—C24—C25—C26	1.5 (5)
C21—P—C11—C12	−100.1 (2)	C24—C25—C26—C21	0.1 (5)
Pd—P—C11—C12	131.93 (19)	C22—C21—C26—C25	−1.5 (4)
C16—C11—C12—C13	−1.0 (4)	P—C21—C26—C25	179.7 (2)
P—C11—C12—C13	177.4 (2)	C11—P—C31—C36	90.4 (2)
C11—C12—C13—C14	−1.0 (4)	C21—P—C31—C36	−161.3 (2)
C12—C13—C14—N	−179.3 (3)	Pd—P—C31—C36	−32.1 (3)
C12—C13—C14—C15	1.4 (4)	C11—P—C31—C32	−86.0 (2)
N—C14—C15—C16	−179.0 (3)	C21—P—C31—C32	22.3 (2)
C13—C14—C15—C16	0.2 (4)	Pd—P—C31—C32	151.50 (19)
C14—C15—C16—C11	−2.3 (4)	C36—C31—C32—C33	−0.4 (4)
C12—C11—C16—C15	2.7 (4)	P—C31—C32—C33	176.1 (2)
P—C11—C16—C15	−175.8 (2)	C31—C32—C33—C34	−0.8 (4)
C13—C14—N—C2	168.6 (3)	C32—C33—C34—C35	0.9 (5)
C15—C14—N—C2	−12.2 (4)	C33—C34—C35—C36	0.2 (5)
C13—C14—N—C1	7.0 (4)	C34—C35—C36—C31	−1.4 (5)
C15—C14—N—C1	−173.8 (3)	C32—C31—C36—C35	1.5 (4)
C11—P—C21—C26	178.4 (2)	P—C31—C36—C35	−175.0 (2)

Symmetry codes: (i) −*x*+1, −*y*+1, −*z*+1.

### Hydrogen-bond geometry (Å, °)

**Table:**

*D*—H···*A*	*D*—H	H···*A*	*D*···*A*	*D*—H···*A*
C25—H25···Cl^ii^	0.95	2.79	3.710 (3)	163
C26—H26···Cl	0.95	2.74	3.159 (3)	108
C36—H36···Cl^i^	0.95	2.77	3.529 (3)	138

Symmetry codes: (ii) −*x*+1, −*y*, −*z*+1; (i) −*x*+1, −*y*+1, −*z*+1.
